# Characterization of the *PHO1* Gene Family in *Vigna radiata* L. and Its Expression Analysis Under Phosphate-Deficient Stress

**DOI:** 10.3390/genes17010025

**Published:** 2025-12-28

**Authors:** Lina Jiang, Ping Sun, Tingting Zhou, Yang Liu, Zihan Kong, Nan Zhang, Hongli He, Xingzheng Zhang

**Affiliations:** Jilin Provincial Key Laboratory of Plant Resource Science and Green Production, Jilin Normal University, Siping 136000, China

**Keywords:** *Vigna radiata*, *PHO1*, bioinformatics, low-phosphorus stress, expression patterns

## Abstract

**Background:** Phosphorus is an essential nutrient for plant growth and development, playing a multifaceted and vital role in plants. Phosphate Transporter 1 (PHO1) is a class of important functional genes involved in plant phosphorus uptake and transport. We identify *PHOSPHATE 1* (*PHO1*) members in mung beans and investigate their response to low phosphorus stress, thereby aiding in the development of stress-tolerant, high-yielding mung bean varieties. **Methods:** A bioinformatic analysis was performed, which led to the identification of the *PHO1* homologue sequence in mung beans. This analysis also elucidated its gene and protein structural characteristics alongside its phylogenetic relationships. qRT-PCR was used to analyze the expression patterns of genes in roots and leaves in response to conditions of prolonged low-phosphorus and phosphorus-deprivation stress. **Results:** Total *PHO1* homologues were identified in mung beans, which can be grouped into 3 groups (Group I-III). Phylogenetic analysis indicates that *VrPHO1*s shares closer evolutionary relationships with *PHO1* in legumes, and exhibits 6 collinear gene pairs with *Glycine max* (soybean), all with *Ka/Ks* ratios below 1, suggesting they have undergone purifying selection. The gene promoter region contains multiple cis-acting elements capable of participating in plant growth and development, stress responses, and plant hormone responses. Expression analysis revealed that more *VrPHO1* genes responded to phosphorus stress in roots than in leaves; of these, the expression of *VrPHO1; H2*, *VrPHO1; H3*, and *VrPHO1; H5* genes was significantly induced by continuous phosphorus-deficient stress. **Conclusions:** This study provides a comprehensive genome-wide identification of the *PHO1* family in mung bean and provides valuable candidate gene resources for the future study of their biological functions and regulatory roles in phosphate-deficient stress.

## 1. Introduction

*Vigna radiata* (L.) Wilcczek (mung bean) are one of China’s important staple crops, being rich in nutrients including starch, protein, dietary fiber, and other essential nutrients for the human body [1]. Compared to other crops, research into the quality of mung beans and their responses to stress remains relatively limited. This situation has directly impeded the rapid advancement of breeding for new mung bean varieties and hindered significant improvements in breeding efficiency, thereby substantially constraining the further expansion and deepening of the mung bean industry [2,3].

Phosphorus (P), as one of the essential nutrients for plants, is extensively involved in biological processes such as the regulation of plant energy metabolism, the construction of cellular structures, and the control of growth and development. The efficient uptake and transport of phosphorus are of paramount importance for plants to complete a series of normal biological processes [4]. Phosphorus in soil primarily exists in organic and inorganic forms, with plant roots efficiently absorbing and transporting inorganic soluble phosphorus [5]. Inorganic phosphorus occurs in low concentrations within soil, whilst organic phosphorus requires conversion before it can be effectively utilized by plants. A deficiency of available phosphorus frequently becomes one of the key stresses limiting the normal growth, development, and yield of plants [3]. Phosphorus deficiency impairs plants’ photosynthetic efficiency, water uptake capacity, and the absorption and utilization of other nutrients [6]. Plants exhibit various symptoms such as pale foliage, stunted growth, and purple discoloration of leaves under conditions of phosphorus deficiency. Concurrently, root development is inhibited, with a marked reduction in the number of secondary roots. In severe cases, plant growth ceases entirely [7]. Moreover, low phosphorus levels may also cause delayed flowering and ripening in *Hordeum vulgare* (wheat), resulting in smaller ears and reduced grain filling, ultimately leading to lower yields and poorer quality [8].

The uptake and transport of phosphorus by plants is primarily achieved through various families of phosphate transporters, Phosphate transporters in plants may be further categorized into high-affinity and low-affinity types based on their affinity for phosphate [5,9]. Currently, phosphate transporters are primarily categorized into six classes: PHT1, PHT2, PHT3, PHT4, PHT5, and PHO1 [10,11,12]. Research has revealed that the PHO1 protein possesses SPX and EXS domains at its N-terminal and C-terminal ends, respectively. The SPX domain functions as a phosphate signal transducer, whilst the EXS domain participates in phosphate transmembrane transport by forming a phosphate transport channel [13]. The PHO1 protein is involved in the uptake and distribution of phosphate, facilitating the transport of phosphorus absorbed by the roots to the aerial parts of the plant. This promotes the transfer of phosphorus from the roots to the stem and leaf tissues [14]. Moreover, PHO1 also participates in the regulation of plant phosphorus homeostasis and responses to low-phosphorus stress [9].

To date, 11 [15], 12 [5], 3 [5], and 14 [5] *PHO1* homologues have been identified in *Arabidopsis thaliana*, wheat, *Oryza sativa* (rice), and soybean, respectively, with their functions having been extensively characterized across multiple species [8,15,16]. *AtPHO1*, *AtPHO1; H1* and *AtPHO1; H10* exhibit distinct low-phosphorus response characteristics. Furthermore, sucrose upregulates *AtPHO1* and *AtPHO1; H1* expression under both phosphorus-sufficient and phosphorus-deficient conditions, whilst suppressing *AtPHO1; H10* expression, demonstrating the synergistic regulation between carbon and phosphorus signaling pathways [17]. *TaPHO7* and *TaPHO8* expression levels were significantly elevated under low-phosphorus stress [18], *OsPHO1; 2* is strongly induced under low-phosphorus conditions and has been identified as a key gene for rice tolerance to low phosphorus and yield enhancement [19]. The expression of *HvnPHO1; H2* in roots is induced by low phosphorus, suggesting that this gene plays a key role in the response mechanism to low-phosphorus stress and may be crucial for barley phosphorus metabolism and tolerance to low-phosphorus stress [20]; this expression pattern is similar to that of the rice *OsPHO1-2* genes [9]. The soybean *PHO1* gene family exhibits root-priority low-phosphorus response. It is evident that the *GmPHO1; H7* gene plays a crucial role in the process of phosphorus transport. Moreover, it has been observed that the expression of this gene is significantly induced by low-phosphorus stress. It is imperative to note that this process plays a critical role, especially in root tissues. The activation of this gene has been demonstrated to effectively enhance phosphorus levels in plant roots, thereby improving phosphorus uptake and utilization efficiency [21], *GmPHO1; H9* exhibits high homology with *AtPHO1; H9* and *FaPHO1; H9*, with upregulation of expression in roots and leaves under low phosphorus stress [22]. Researchers discovered that the forest strawberry *FvPHO1; H9* gene is expressed when the plant is under low-phosphorus stress [23]. GUS histochemical staining revealed strong expression signals of the heterologous expressed *TaPHO1; H2* gene were observed in the xylem and leaf vein tissues of *Arabidopsis* plants. Functional analysis revealed that this gene plays a crucial role in the long-distance transport and precise distribution of phosphorus within plants [8].

Despite the *PHO1* gene family being the focus of several studies across a variety of species, research on the mung bean *PHO1* gene remains limited. This study focuses on investigating the composition of the *PHO1* gene family within the mung bean genome, along with its phylogenetic relationships with other plants, such as *Arabidopsis* and soybean. Additionally, it explores whether the members of the gene family demonstrate a response to low-phosphorus stress. It is anticipated that this study will provide valuable insights into the composition of the mung bean *PHO1* gene family and facilitate the discovery of key candidate functional genes that are able to respond to continuous low-phosphorus stress. This research is expected to yield essential components for the breeding of mung beans that possess enhanced phosphorus uptake efficiency.

## 2. Materials and Methods

### 2.1. Experimental Materials and Procedures

This experiment was conducted at the Key Laboratory of Plant Resource Science and Green Production of Jilin Province, Jilin Normal University (E. 124.34515, N. 43.14635). “Maolv No. 1”, a mung bean cultivar bred in this laboratory with plump and uniform seeds, was selected as the experimental material, and the sand culture method was adopted throughout the study.

Seed germination was induced via the water agar germination method: seeds were neatly arranged in culture trays with their germinal end facing downward on moistened filter papers. Following 3 days of incubation, seedlings with uniformly developed root systems and fully expanded cotyledons were transplanted into 70 × 80 mm seedling pots filled with 0.5–1 mm sand particles (pre-rinsed thoroughly with distilled water to remove potential phosphorus contamination). The pots were then placed in a constant-temperature incubator under controlled conditions: 23 °C, light intensity of 200 μmol m^−2^ s^−1^, a 16 h light/8 h dark photoperiod, and 80% relative humidity. Seedlings with consistent growth were selected for experimental treatments, which included three replicates (10 plants per replicate). Modified one-half Hoagland’s nutrient solution (pH 6.0) was used as the base nutrient medium, with phosphate (Pi) concentration adjusted by modifying the amount of KH_2_PO_4_. KCl was supplemented to low-phosphorus nutrient solutions to balance K^+^ concentration across low-phosphorus treatments, and the nutrient solution was replenished every 2 days to ensure adequate nutrient supply. Based on previous research [24], Pi concentration gradients were established according to the range of mung bean responses to low-phosphorus stress: normal phosphorus supply, phosphorus deficiency (0P, 0 μM Pi), and low-phosphorus stress (2.5 μM, 10 μM, 50 μM Pi). Preliminary experiments demonstrated that this cultivar exhibited the most pronounced stress response phenotype at 10 μM Pi, with leaf wilting observed on the 8th day of treatment. Thus, 0 μM and 10 μM Pi were designated as the phosphorus deficiency (0P) and low-phosphorus (LP) treatments, respectively, while 500 μM Pi served as the normal phosphorus (NP) control.

Treatments were initiated when the first trifoliolate leaf was fully expanded, and the experiment lasted 8 days to investigate the gene expression patterns in response to continuous low-phosphorus stress. Root and leaf samples were collected every 2 days, and immediately after collection, samples were flash-frozen in liquid nitrogen and stored at −80 °C for subsequent RNA extraction and expression analysis. To minimize the impact of individual variation, roots from three individual plants were pooled into one replicate. Furthermore, three independent biological replicates were established for each treatment condition.

### 2.2. Identification and Physicochemical Characterization of Members of the Mung Bean PHO1 Gene Family

Based on published literature on the *PHO1* gene family in *A. thaliana*, *Oryza sativa*, *Triticum aestivum*, and *Glycine max*, relevant data were retrieved from databases including TAIR (https://www.arabidopsis.org/ (accessed on 7 July 2025)), Gramene (https://www.gramene.org/ (accessed on 9 July 2025)), Ensembl Plants (http://plants.ensembl.org/index.html (accessed on 10 July 2025)), Phytozome database (https://phytozome-next.jgi.doe.gov/ (accessed on 13 July 2025)), and the Legume Information System (LIS, https://www.legumeinfo.org/ (accessed on 20 July 2025)). Candidate homologous genes were identified through homology-based alignment. Conserved domains in the candidate proteins were predicted using the NCBI Conserved Domain Database (CDD, https://www.ncbi.nlm.nih.gov/ (accessed on 2 November 2025)) and InterPro (https://www.ebi.ac.uk/interpro/ (accessed on 10 December 2024)). Duplicated sequences and those with incomplete domains were manually removed, leading to the identification of the mung bean *PHO1* gene family members. The physicochemical properties of the proteins were predicted using the Expasy platform, with an e-value threshold of 0.0001. Subcellular localization was predicted using WoLF PSORT (https://wolfpsort.hgc.jp/ (accessed on 17 March 2025)).

### 2.3. Structural Analysis of the PHO1 Genes and Protein in Mung Beans

Based on the mung bean genome annotation file (GFF3 format), a physical map of gene chromosomal locations was constructed using TBtools-II (v2.2). The tertiary structures of the corresponding proteins were predicted using Expasy (https://www.expasy.org/ (accessed on 28 May 2025)). Conserved motifs were identified with the online tool MEME (https://meme-suite.org/meme/index.html (accessed on 20 March 2025)), where the number of motifs was set to 15 and all other parameters were kept as default. Gene structures and conserved motifs were subsequently analyzed and visualized using TBtools-II (v2.2).

### 2.4. Systematic Evolution and Collinearity Analysis of the PHO1 Gene Family in Mung Bean

To elucidate the evolutionary relationships and divergence levels among mung bean *PHO1* gene family members, multiple sequence alignment was performed using Clustal W implemented in MEGA software (v11.0.13). Subsequently, a phylogenetic tree was constructed for PHO1 proteins from five species—soybean, wheat, rice, *A. thaliana*, and mung bean—employing the neighbor-joining method with 1000 bootstrap replicates. To visualize phylogenetic relationships, the resulting tree was annotated using the iTOL (https://itol.embl.de/ (accessed on 2 November 2025)) web platform. Based on genome annotation files (GFF3 format) for *A. thaliana*, mung bean, and soybean, TBtools-II (v2.2) was utilized to analyze both intra- and inter-specific collinearity relationships, enabling identification of syntenic gene pairs. The selection pressure acting on these gene pairs was assessed by calculating Ka/Ks ratios, with divergence time estimated using the molecular clock formula *T = Ks/2λ* (λ = 6.1 × 10^−9^) [25].

### 2.5. Analysis of Cis-Acting Elements in the PHO1 Genes Promoter of Mung Beans

The 2000 bp region upstream of the transcription start site was defined as the promoter sequence for analysis. Using TBtools-II (v2.2), the Gtf/Gff3SequencesExtract function was employed to process the mung bean GFF3 annotation file alongside the genomic sequence file. Subsequently, the promoter sequences (2000 bp in length) were extracted using the Fasta Extract function. The extracted sequences were then submitted to the Plant CARE online database (https://bioinformatics.psb.ugent.be/webtools/plantcare/html/ (accessed on 21 March 2025)) for the identification of cis-acting regulatory elements, and the results were visualized using its ‘Basic Bio sequences View’ functionality.

### 2.6. Gene Expression Analysis and Data Processing

To investigate the expression patterns of Maolv No. 1 across various tissues and under low-phosphorus stress, gene-specific primers were designed using NCBI Primer-BLAST (https://www.ncbi.nlm.nih.gov/tools/primer-blast/index.cgi (accessed on 31 July 2025)) based on the cDNA sequence extracted from mung bean genome (VC1973A.gnm7) in LIS. qRT-PCR was conducted on a Light Cycler 96 system (Roche, Switzerland), and the *VrTUA* gene was used as an internal control [26]. Each 20 μL reaction contained 10 μL of 2× Ultra SYBR Mixture (CWBIO, China), 0.4 μL each of forward and reverse primers (10 mmol/L), 1.5 μL of cDNA template, and 7.7 μL of ddH_2_O. The RT-qPCR procedure was as follows: 94 °C for 3 min followed by 40 cycles at 94 °C for 15 s, 60 °C for 15 s and 72 °C for 30 s. Primer specificity was confirmed by examining amplification and melting curves. The primers used for qRT-PCR are listed in Supplementary Materials Table S2. Three biological replicates were included for each sample. Relative gene expression levels were calculated using the 2^(–ΔΔCT)^ method, and standard deviations were determined. Each experiment was conducted with three biological and three technical replicates. Statistical analysis was performed using a two-tailed Student’s *t* test in SPSS Statistics for Windows, Version 23.0 software.

## 3. Results and Analysis

### 3.1. Identification of Members of the Mung Bean PHO1 Gene Family and Analysis of Their Protein Physicochemical Properties

Using bioinformatics approaches, five members of the *PHO1* gene family were identified in mung bean (Table 1). These were designated *VrPHO1; H1* to *VrPHO1; H5* according to their chromosomal locations. The analysis revealed that the amino acid length of the PHO1 proteins ranges from 506 aa (VrPHO1; H1) to 791 aa (VrPHO1; H2), with molecular weights between 58.68 and 92.02 kDa and theoretical isoelectric points (pI) ranging from 9.05 to 9.55. Therefore, all VrPHO1 family members are predicted to be alkaline proteins (pI > 7). Additionally, all five proteins show negative grand average of hydrophobicity (GRAVY) values, indicating substantial hydrophilicity and classifying them as hydrophilic proteins. The instability index ranged from 36.56 to 46.66. Among them, only VrPHO1; H1 displayed an instability index below 40 and was predicted to be stable, while the other four proteins exhibited instability indices above 40 and were categorized as unstable. The aliphatic index ranged from 88.64 to 94.29, suggesting that proteins in the VrPHO1 family generally possess high thermostability, with higher values indicating greater structural flexibility.

### 3.2. Chromosomal Localization Analysis of the PHO1 Gene Family in Mung Beans

**Figure 1:**
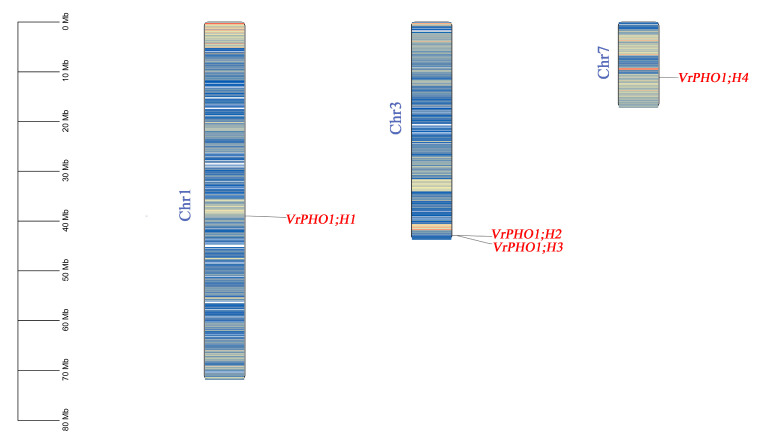
Chromosomal localization analysis of *VrPHO1* gene family members. Different colors represent gene density.

We isolated and chromosomally mapped five members of the mung bean *PHO1* gene family (Figure 1). Specifically, four genes localize to chromosomes 1, 3, and 7, while *VrPHO1; H5* resides on scaffold 62. This uneven chromosomal distribution typically stems from evolutionary events such as gene duplication and chromosomal rearrangement, offering critical insights into the evolutionary dynamics and functional regulation of the *PHO1* gene family in mung.

### 3.3. Structural Analysis Prediction of VrPHO1s Proteins

Secondary structure analysis of mung bean PHO1 proteins via SOPMA revealed that all VrPHO1 members consist of α-helices, β-sheets, and random coils. Among these structural components, α-helices accounted for the highest proportion, with their content ranging from 51.2% (VrPHO1; H5) to 56.32% (VrPHO1; H2). In contrast, β-sheets exhibited relatively low content, varying from approximately 3.95% (VrPHO1; H2) to 5.19% (VrPHO1; H5). The proportion of random coils spanned from 39.72% (VrPHO1; H2) to 43.62% (VrPHO1; H5).

**Figure 2:**
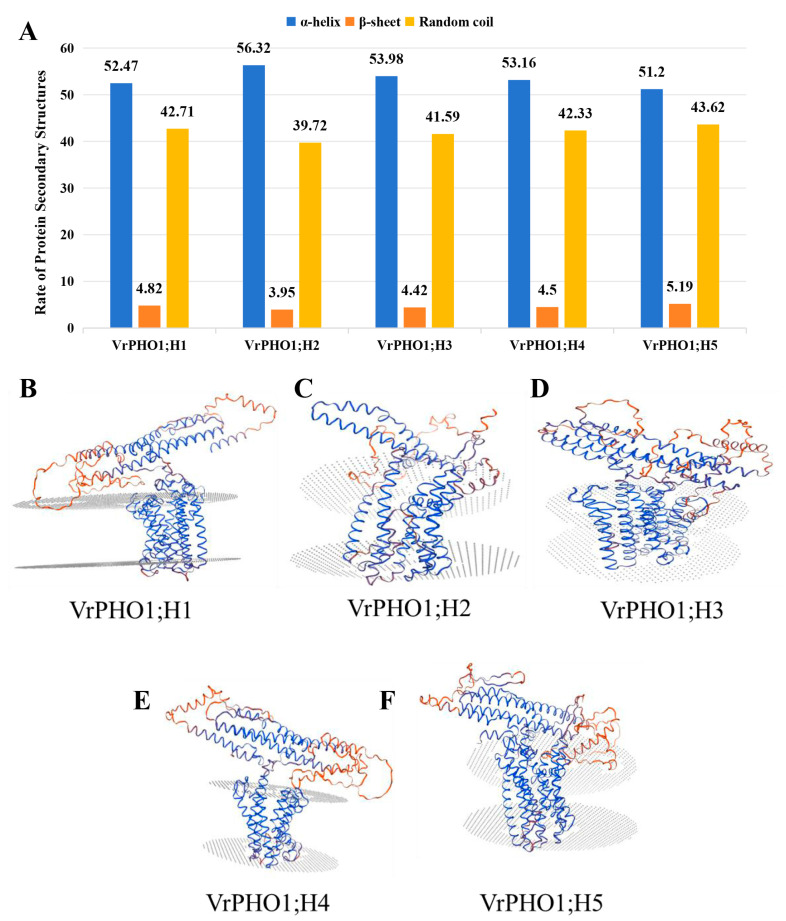
Structural prediction analysis of the VrPHO1s protein. (**A**) Secondary structure of the VrPHO1s protein. (**B**–**F**). Tertiary structure of the VrPHO1s protein.

These prediction results were consistent with the evolutionary relationships among VrPHO1 members (Figure 2A), wherein more closely related members exhibited higher similarity in secondary structure. Tertiary structure prediction (Figure 2) showed that VrPHO1 proteins contain an SPX domain at the N-terminus and an EXS domain at the C-terminus, respectively, and adopt a typical α-helical folding pattern.

### 3.4. Phylogenetic Tree Analysis of the VrPHO1 Gene Family Members

**Figure 3:**
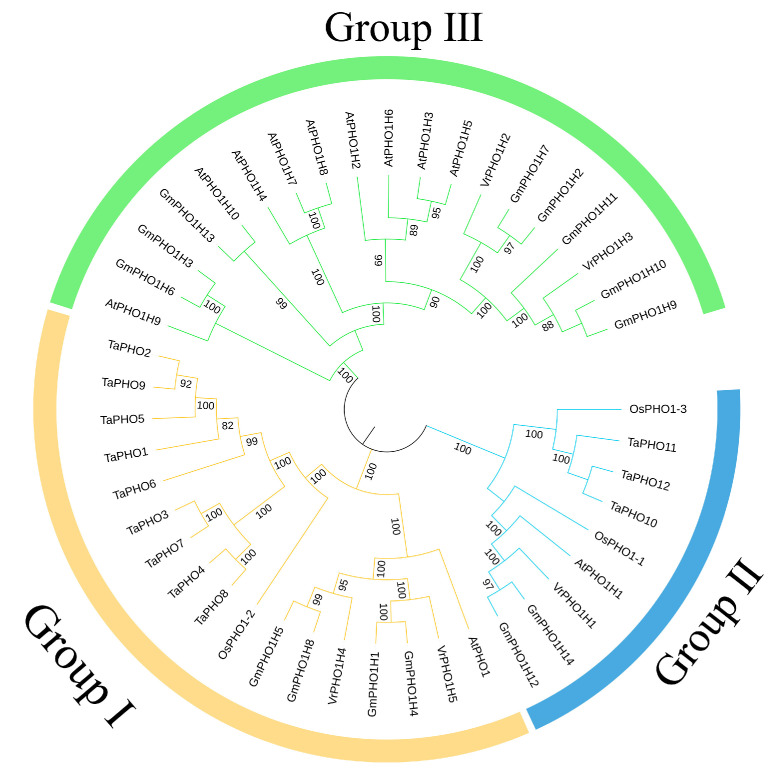
Phylogenetic tree of *PHO1* gene family members across species.

The evolutionary relationships among members of the mung bean *PHO1* gene family were determined through the alignment of PHO1 proteins from *A. thaliana*, soybean, wheat, and rice with their corresponding mung bean proteins. This alignment was facilitated by the use of MEGA software (v11.0.13). A phylogenetic tree was subsequently constructed (Figure 3). The results indicate that the mung bean *PHO1* gene family could be classified into three subfamilies (Group I, Group II, and Group III). Group II is distinguished by its minimal membership, comprising solely *VrPHO1; H1*. In contrast, Groups I and III each comprise two members. Specifically, *VrPHO1; H4* and *VrPHO1; H5* are situated in Group I, while *VrPHO1; H2* and *VrPHO1; H3* are located in Group III. Moreover, the study demonstrated that the *VrPHO1* genes predominantly clusters with the soybean *PHO1* genes on the same phylogenetic branch. This finding provides crucial reference points and theoretical foundations for in-depth investigations into the structure and function of the mung bean *PHO1* gene family.

### 3.5. Genetic Structure and Analysis of Conserved Protein Motifs in Members of the VrPHO1 Gene Family

Members of the same gene family typically exhibit distinct differences in gene and protein structures. Gene structure analysis showed that the number of exons in *VrPHO1* members ranged from 12 (*VrPHO1; H2*) to 15 (*VrPHO1; H4*). Conserved domain analysis via Pfam and NCBI databases confirmed that all VrPHO1 members contain SPX and EXS domains—key functional domains of PHO1 proteins.

**Figure 4:**
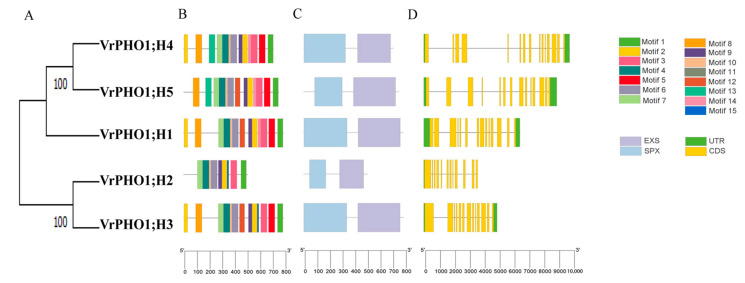
Conserved motifs, protein conserved domains and gene structure analysis of *VrPHO1* gene family members. (**A**) Intraspecific phylogenetic tree of *VrPHO1* genes. (**B**) Conserved motifs of the VrPHO1 protein, with squares of different colors representing distinct motifs. (**C**) Conserved domains of the VrPHO1 protein, with squares of different colors representing distinct protein domains. (**D**) *VrPHO1* genes’ structure.

Conserved motif analysis identified 15 conserved motifs across the five VrPHO1 proteins (Figure 4). Notably, VrPHO1; H2 contained only 9 motifs, while the other four members each had 13 motifs—this discrepancy aligns with its more distant phylogenetic relationship with other members. Although VrPHO1; H1 (Group II) and VrPHO1; H3 (Group III) belong to different subfamilies, all five VrPHO1 proteins shared 10 conserved motifs (Motif 1 to Motif 10). This motif conservation corresponds to the conserved functional domains and is closely associated with the core function of PHO1 proteins.

Consistently, conserved domain prediction for the five VrPHO1 proteins showed that all contained both EXS and SPX domains, with the SPX domain located at the N-terminus and the EXS domain at the C-terminus.

### 3.6. Analysis of Cis-Acting Elements in the VrPHO1 Genes Promoter

**Figure 5:**
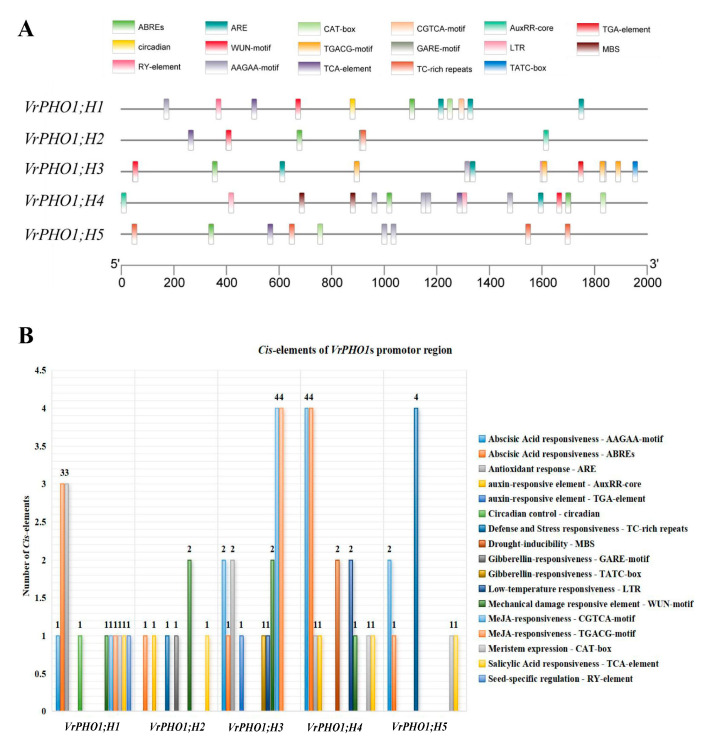
Structure of promoter cis-acting elements associated with hormones, stress responses and growth development in members of the *VrPHO1* gene family. (**A**) Distribution of cis-acting elements in the *VrPHO1* genes promoter region, with squares of different colors representing distinct elements. (**B**) Distribution of cis-acting elements within the *VrPHO1* genes promoter region, with squares of different colors representing distinct functions.

In order to gain deeper insight into the function of the *VrPHO1s*, a sequence spanning 2000 bp upstream of the start codon of the mung bean *PHO1* gene was extracted and uploaded to the Plant CARE website for analysis (Figure 5). The aim of this analysis was to identify key cis-acting elements. The analysis revealed that all five genes collectively contain 17 light signal response elements. Of these, 10 types comprise plant hormone (auxin, salicylic acid, abscisic acid, jasmonic acid, and gibberellin) response elements. In the field of stress biology, the study of response elements to abiotic stress (i.e., low temperature, drought, and mechanical injury) has been a subject of considerable research interest. Notable examples of such elements include GARE-motif, ABREs, and AuxRR-core elements. In addition, four elements have been identified as responding to abiotic stress (i.e., cold, drought, and mechanical injury), namely, WUN-motif, MBS, and LTR. Furthermore, five elements have been implicated in growth and development (i.e., related to circadian rhythms, seed specificity, and meristem expression), including circadian and CAT-box elements. Of these, *VrPHO1; H2* contains the fewest light response elements at six, while *VrPHO1; H4* contains the most at fifteen. The remaining genes contain eleven, thirteen, and nine, respectively. An analysis of cis-acting elements within the promoter regions of five *VrPHO1* genes was conducted, which revealed the presence of multiple elements associated with environmental stress and hormone responses. The elements in question include the drought response element MBS, the abscisic acid response element ABRE, the auxin-responsive element TGA, the low-temperature response element LTR, and elements involved in defense and stress responses.

### 3.7. Inter-Species and Intra-Species Collinearity Analysis of the VrPHO1 Genes

**Figure 6:**
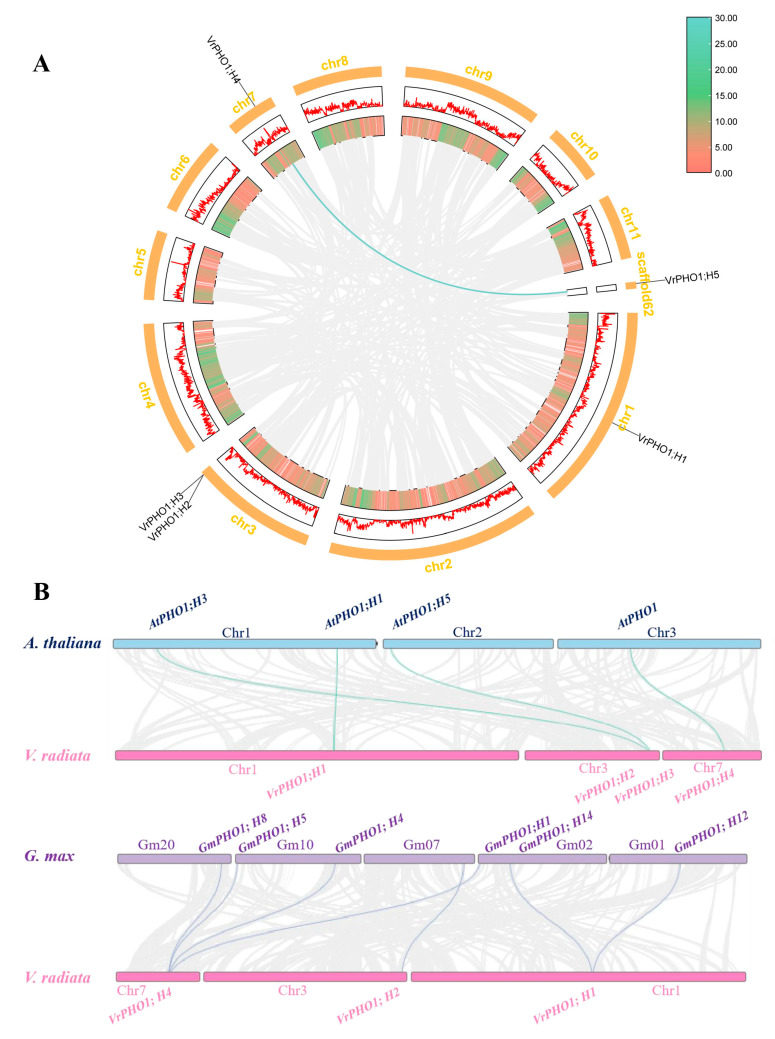
Collinearity analysis of *VrPHO1* gene family members. (**A**) Intraspecific collinearity of the *VrPHO1* genes. (**B**) Collinearity of mung bean *PHO1* family genes with other species.

In order to ascertain the evolutionary relationships of the *VrPHO1* genes, an inter-genomic analysis was conducted using TBtools-II software (v2.2). (Supplementary Materials Table S2) Within the species, only a single pair of collinear genes was identified: *VrPHO1; H4/VrPHO1; H5* (Figure 6A). An interspecific synteny analysis of the *PHO1* gene families in mung beans and soybeans yielded the results illustrated in Figure 6, thereby identifying seven pairs of homologous genes (Figure 6B). It was established that two pairs are present between *VrPHO1; H1* and *GmPHO1; H1*, and between *VrPHO1; H1* and *GmPHO1; H14*, respectively. Furthermore, four pairs were identified between *VrPHO1; H2/GmPHO1*; *H2, VrPHO1; H4/GmPHO1; H1, GmPHO1; H4, GmPHO1; H5,* and *GmPHO1; H8*. The identification of four pairs of collinear genes (*VrPHO1; H1/AtPHO1; H1, VrPHO1; H2/AtPHO1; H3, VrPHO1; H3/AtPHO1; H5*, and *VrPHO1; H4/AtPHO1*) was successful in both the mung bean and the *A. thaliana*.

In order to analyze the selective pressures acting upon genes during evolution, a combination of intraspecific and interspecific collinearity analyses was employed. This approach enabled the estimation of the non-synonymous substitution rate (*Ka*), the synonymous substitution rate (*Ks*), and the *Ka/Ks* ratio for gene pair members. The results presented in Table 2 demonstrate that the *Ka/Ks* ratios for all gene pairs were below 1.0, indicating that their *VrPHO1* genes have undergone negative selection, also known as purifying selection. Furthermore, the divergence time for the five *VrPHO1* genes in mung beans is estimated to be approximately 32 to 107 million years ago (MYA).

### 3.8. Analysis of VrPHO1 Genes Expression Patterns Under Low-Phosphorus Stress

To characterize the mung bean *PHO1* gene family’s transcriptional response to low-phosphorus stress, we used qRT-PCR to profile *VrPHO1* expression in roots and leaves under 0P, LP, and NP conditions. Results revealed striking gene-specific and time-dependent dynamics.

**Figure 7:**
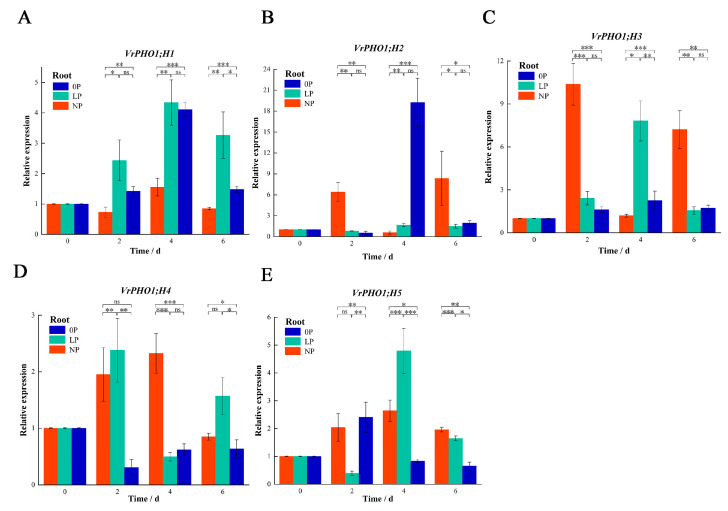
Analysis of *VrPHO1* gene family members’ expression levels in roots. (**A**–**E**) Relative expression levels of *VrPHO1; H1–VrPHO1; H5* at 0P, LP, and NP concentrations. Data significantly different from the corresponding controls (NP) are indicated (* *p* ≤ 0.05; ** *p* ≤ 0.01; *** *p* ≤ 0.001; ns > 0.05; Student’s *t* test). Statistical methods of images were always set the same within experiments.

In roots (Figure 7): *VrPHO1;H1* showed stage-specific LP dominance (significantly higher at 2–4 days vs. 0P/NP, attenuating by day 6); *VrPHO1;H2* had 0P-specific induction only at day 4 (expression drastically higher vs. other groups); *VrPHO1; H3* exhibited dynamic reversal (NP-dominant at day 2, LP-upregulated by day 4); *VrPHO1; H4* displayed early-stage differences (LP-upregulated at day 2, 0P sharply reduced at day 4).

**Figure 8:**
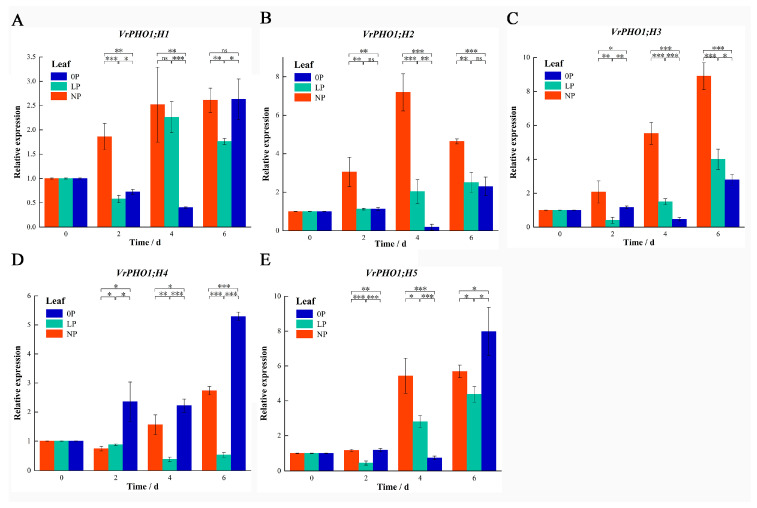
Analysis of *VrPHO1* gene family members’ expression levels in leaves. (**A**–**E**) Relative expression levels of *VrPHO1; H1–VrPHO1; H5* at 0P, LP, and NP concentrations. Data significantly different from the corresponding controls (NP) are indicated (* *p* ≤ 0.05; ** *p* ≤ 0.01; *** *p* ≤ 0.001; ns > 0.05; Student’s *t* test). Statistical methods of images were always set the same within experiments.

In leaves (Figure 8): *VrPHO1; H1* had stage-dependent shifts (NP-dominant at day 2, LP-surpassed by day 4); *VrPHO1; H2* showed NP-dominant induction at day 2 (drastically higher vs. others); *VrPHO1; H3* sustained NP dominance throughout; *VrPHO1; H4* had mid-stage NP dominance (drastically higher at day 4, 0P persistently low); *VrPHO1; H5* exhibited fluctuating alternation in both tissues.

These regulatory features demonstrate the functional differentiation of *PHO1* in metabolism under phosphorus stress, providing crucial evidence for the cooperative mechanisms underpinning plant phosphorus homeostasis.

## 4. Discussion

Phosphorus is an essential element for crop growth. When phosphorus deficiency occurs, plants exhibit adverse traits such as stunted growth and leaf curling, severely impacting normal plant development and high-quality agricultural production [27]. Due to the low content of available phosphorus in soil, phosphorus often becomes a limiting factor in plant growth and development. Enhancing plants’ capacity for phosphorus uptake and transport is therefore a significant research topic. The *PHO1* gene family constitutes one of the core genes regulating phosphorus transport, primarily involved in phosphorus transport and homeostasis maintenance. To date, members of this gene family have been identified in key plant species including *A. thaliana*, *Tribulus terrestris*, soybean, rice, maize and oilseed rape, with significant variation observed in the number of family members present.

### 4.1. Evolutionary Analysis of PHO1 Genes Exhibited Specificity Among Diverse Plants

In ancient polyploid species, genes can be classified into three distinct categories: WGD-derived genes, small-scale duplication genes, and single-copy genes [28]. Whole-genome duplications (WGDs) and polyploidy are key mechanisms in the formation of gene families and play a crucial role in the functional and evolutionary processes of plant genomes. The Leguminosae family of plants first appeared approximately 67 million years ago (MYA) [29,30] The *Papilionoideae* clade emerged most recently, at approximately 58 MYA. Subsequently, approximately 20 million years ago, a divergence occurred between *Hologalegina* and *Phaseoloid*. *Hologalegina* represents the modern cool-season legumes, including Medicago, peas, and *Lotus japonicus*, while *Phaseoloid* encompasses the warm season legumes, such as soybean, common bean, and mung bean [31,32]. During the process of divergent evolution, mung beans experienced only the whole-genome duplication event shared by all legumes [33], resulting in a genome smaller than that of soybeans. This study screened and identified five *PHO1* homologue sequences in mung beans based on homology alignment and conserved domain annotation. It is significantly fewer than the number of gene family members identified in soybeans. This phenomenon is intimately associated with the intricacy of the genome. Furthermore, seven pairs of collinear gene pairs were yielded by the use of mung bean and soybean for collinearity analysis. The *Ka/Ks* ratios for homologous gene pairs within the *PHO1* family of mung beans and soybeans ranged between 0.14 and 0.36, all less than 1. This implies that these genes have undergone purifying selection during evolution, suggesting that their functions have remained relatively unchanged. Estimated divergence times ranged from 32.92 to 107.23 MYA, revealing significant divergences. The gene pairs *GmPHO1; H14/VrPHO1; H1* (32.92 MYA) and *GmPHO1; H12/VrPHO1; H1* (36.22 MYA) exhibited relatively recent divergence times and closer phylogenetic relationships. By contrast, *GmPHO1; H4/VrPHO1; H4* (107.23 MYA) and *GmPHO1; H1/VrPHO1; H4* (106.61 MYA) diverged earlier, with longer separation times, reflecting distinct evolutionary patterns during species divergence and gene family expansion. This is associated with soybean undergoing at least two whole-genome duplications, resulting in genome doubling. Furthermore, due to limitations in the completeness of the mung bean genome assembly employed, *VrPHO1; H5* has not been localized to any known specific chromosome either. As research into the mung bean genome deepens, it will yield increasingly rich information. The physical distance between the *VrPHO1; H2* and *VrPHO1; H3* genes on chr3 is considerably less than 200 kb, suggesting that they may have originated from tandem duplication. However, the present study reveals certain structural differences between the *VrPHO1; H2* and *VrPHO1; H3* genes and proteins, which may result from structural variations arising after duplication. *VrPHO1; H2* exhibits a lack of individual motifs. However, it should be noted that all other members of the *PHO1* family retain complete motifs in mung bean, characterized by highly consistent protein domains and gene configurations within the same subfamily.

On the basis of phylogenetic analysis, structural characteristics, and functional differentiation, a definitive classification into two evolutionary lineages (Class I and Class II) can be proposed [34]. These clades have evolved distinct functional roles throughout the process of plant evolution. Class II represents the ancestral lineage, having formed earlier than plant diversification into terrestrial and aquatic species. In contrast, Class I constitutes the derived lineage, which is believed to have originated from functional differentiation following an early whole-genome duplication event. The monocotyledonous *PHO1* family exclusively comprises Class II members. Research indicates that Class I participates in responses to stresses such as high salinity, while Class II is involved in phosphorus homeostasis and its long-distance transport from roots to aboveground parts. Furthermore, Class II genes have been also shown to exhibit more stable expression patterns. These patterns are predominantly constitutive, yet they remain subject to regulation by phosphorus starvation [35]. Phylogenetic analysis in this study revealed that *PHO1* homologues are divided into three groups: Group III (*VrPHO1; H2/H3*) corresponds to Class I, while Groups I (*VrPHO1; H4/H5*) and Group II (*VrPHO1; H1*) correspond to Class IIA and Class IIB, respectively.

### 4.2. Functional Conservation and Diversification of VrPHO1 Genes in Mung Beans

The results of the research indicate that the PHO1 protein is mainly located on the plasma membrane, where it is responsible for the transportation of Pi from the intracellular to the extracellular area. The subcellular localization prediction results of the present study corroborate this perspective. The PHO1 protein is a core component that plays a crucial role in regulating plant phosphorus homeostasis, and its functional stability and specificity are derived from the conservation and differential modification of its SPX-EXS dual domains. The SPX domain is responsible for sensing intracellular phosphorus signals, while the EXS domain, which contains multiple transmembrane helices, facilitates phosphate transport across membranes. The highly conserved SPX1-3 subdomains, IP_6_-binding cavity, and dimerization within the SPX domain ensure precise intracellular phosphorus signal perception across diverse plant PHO1 proteins [36,37]. Concurrently, the sequence conservation of Trp/Tyr residues and phosphate-binding sites within the EXS domain maintains the core activity of transmembrane transport [37]. This structural conservation is widely observed across the plant taxa of mosses to angiosperms, reflecting its evolutionary necessity for adapting to phosphorus-limited environments following the process of plant territorialization. Domain differentiation also constitutes the core driver of functional diversification within the *PHO1* family [37]. It has been demonstrated that sequence variation within the SPX domain differentiates the response thresholds and kinetic properties of PHO1 proteins across species in response to phosphate signals. Concurrently, subtle alterations in the length of the transmembrane helices within the EXS domain, and the amino acid composition of the protein’s inner surface, directly influence phosphate transport efficiency and substrate specificity [38]. Furthermore, the low conservation of the SPX-EXS linker region and C-terminal enables species-specific regulation of post-translational modifications (e.g., phosphorylation) and protein interactions in PHO1, thereby expanding functional diversity [37]. This mechanism is pivotal in protecting fundamental phosphate transport while concurrently ensuring evolutionary flexibility for plant adaptation to diverse ecological conditions, including low phosphorus and drought.

Furthermore, *VrPHO1; H4* and *VrPHO1; H5* have been found to belong to the same clade as *AtPHO1*, whereas *VrPHO1; H1* has exhibited an identical clade to that of *AtPHO1; H1*. As demonstrated previously, *AtPHO1* and *AtPHO1; H1* have been shown to play a pivotal role in the process of loading root phosphorus (Pi) into the phloem, thereby facilitating its efflux from the cytoplasm into the phloem vessels and, in turn, enabling the upward transport of Pi from the roots [39]. Structural analysis by cryo-electron microscopy reveals that *AtPHO1; H1* facilitates phosphate efflux via a homomeric channel [37]. Previous results also indicate that *OsPHO1;2* and *GmPHO1; H4*, which share homology with *AtPHO1*, are also involved in phosphorus transport and stress responses [40]. The *PHO1* gene family constitutes one of the core genes regulating phosphorus transport, primarily involved in phosphorus transport and homeostasis maintenance [40]. To date, members of this gene family have been identified in key plant species, including *A. thaliana*, *Cicer arietinum*, soybean, rice, maize and wheat, with significant variation observed in the number of family members present [5,18,40,41]. In soybean, their expression exhibits complex patterns that are regulated by both salt stress and phosphorus starvation. Ectopic expression of *GmPHO1; H6* in *A. thaliana* exhibited consistent performance with the wild type under low-phosphorus conditions, indicating that *GmPHO1; H6* is insensitive to phosphorus stress and that over-expression does not affect phosphorus stress resistance. The present study investigates the impact of the soybean *GmPHO1; H4/H8/H9* gene on plant salt tolerance and adaptation to low-phosphorus stress [35]. The results demonstrate that the over-expression of *GmPHO1; H4/H8/H9* significantly enhances plant salt tolerance while simultaneously improving adaptation to low-phosphorus stress, indicating functional diversity [35]. In this study, *VrPHO1; H4*, which is phylogenetically closely related to the *GmPHO1; H4*, was also significantly induced in leaves under phosphorus-deficient conditions. This study indicates differential expression of *VrPHO1* genes in root and leaf tissues under low-phosphorus and phosphorus-deficient conditions. Roots possess additional genes capable of responding to low-phosphorus or phosphorus-deficiency stress, and *VrPHO1*s are induced in roots by continuous phosphorus-deficiency or low-phosphorus conditions. However, differences exist in the induction time and pattern. Among these, *VrPHO1; H1*, *VrPHO1; H2*, and *VrPHO1; H5* exhibited more pronounced induction under stress conditions. *VrPHO1; H2* and *VrPHO1; H5* responded significantly under phosphorus-deficient conditions, whereas *VrPHO1; H4* showed marked induction in leaves under phosphorus-deficient conditions. Moreover, *VrPHO1; H4* and *VrPHO1; H5* exhibit a colinear relationship, suggesting that their functions are relatively conserved.

*Cis*-acting elements regulate gene expression by binding to transcription factors, thereby influencing the initiation and efficiency of gene transcription. Analysis of the *cis*-acting elements within the promoter region of the *VrPHO1* homologues belonging to Class I and Class II indicates that they contain elements not only involved in stress response, but also associated with plant growth and development, as well as plant hormone responses (such as ERE, ABREs element, etc.). These findings suggest that these genes may also play a regulatory role in plant growth and development, demonstrating their potential functional diversity. Previous results indicated that ethylene enhances phosphorus uptake efficiency by regulating the expression of *AtPHO1; H5* in *A. thaliana* [37]. Moreover, it has been determined that the maintenance of Pi homeostasis during the process of grain filling is of critical importance. The results of the research indicate that *OsPHO1;2* possesses the capacity for phosphorus transport, and that it is involved in the process of long-distance transport between root and stem tissues [9]. It is noteworthy that *OsPHO1;2* mutants exhibit a substantial accumulation of Pi within grains, particularly in endosperm cells, thereby severely inhibiting the enzymatic activity and expression of starch synthesis-related enzymes (AGPase) [19,36,42]. This, in turn, has been demonstrated to impair grain filling, thus indicating its involvement in the molecular regulation of grain filling. Evidence indicates that *ZmPHO1;2a* and *ZmPHO1;2b* display the closest relationship to *AtPHO1* [41,43]. Research findings indicate that maize homologues *ZmPHO1;2* similarly regulate grain filling and phosphorus redistribution in maize [36], suggesting that *PHO1* family protein-mediated grain-filling regulation is highly conserved across cereal crops [19,42]. However, the specific biological functions and molecular regulatory mechanisms of its member genes in mung beans remain unclear at present. Previous studies have revealed that the transcription factor SHR participates in regulating phosphorus allocation from roots to shoots by degrading *AtPHO1;H2* [44], while *AtPHO1;H4* can participate in pollen phosphorus homeostasis regulation and embryo development control under the regulation of transcription factor PHR1 [36], Research in this field also provides important avenues for investigating the molecular mechanisms by which mung bean *VrPHO1s* participate in phosphorus transport and allocation.

## 5. Conclusions

In this study, a total of five *PHO1* homologous sequences were identified in mung beans, distributed across four existing chromosomes and one scaffold. The proteins encoded by VrPHO1s all contain the conserved SPX and EXS domains, exhibiting high structural conservation, though differences exist in the composition of conserved motifs among members. Phylogenetic analysis revealed the presence of three clusters within the *VrPHO1* genes, indicating closer phylogenetic relationships and collinearity with soybean within the legume family. The promoter regions of these genes contain cis-acting elements involved in plant hormone and abiotic stress responses, varying in type and number, suggesting functional diversity among gene members. Gene expression analysis under low-phosphorus stress revealed that the majority of genes in roots responded to low- or deficient-phosphorus stress. However, in leaves, only *VrPHO1; H4* responded to phosphorus-deficiency stress, thus rendering them important candidate functional genes. The findings of this study provide crucial candidate gene elements and a theoretical foundation for research on mung bean phosphorus stress responses.

## Figures and Tables

**Table 1:** Identification of *VrPHO1* gene family members and analysis of their protein physicochemical properties.

Gene	Gene ID	Chromosome Localization	Strand	Number of Amino Acids/aa	Molecular Weight/kD	PI	Aliphatic INDEX	Lipolysis Index	Hydrophobicity	Prediction of Subcellular Localization
*VrPHO1; H1*	*Vradi01g00002120*	38992032.. 38998514	+	789	91.66	9.09	43.69	88.95	−0.193	Cytoplasmic membrane
*VrPHO1; H2*	*Vradi03g00002410*	42859336.. 42862967	+	506	58.68	9.38	36.56	92.65	−0.115	Cytoplasmic membrane
*VrPHO1; H3*	*Vradi03g00002411*	42866638.. 42871577	+	791	92.02	9.55	42.8	91.47	−0.188	Cytoplasmic membrane
*VrPHO1; H4*	*Vradi07g00000873*	11103676.. 11113505	+	711	83.13	9.05	45.21	94.29	−0.173	Cytoplasmic membrane
*VrPHO1; H5*	*VradiU00000886*	564256.. 573220	+	752	86.54	9.09	46.66	88.64	−0.103	Cytoplasmic membrane

**Table 2:** Analysis of evolutionary selection pressure on the *VrPHO1* genes.

Gene1	Gene2	*Ka*	*Ks*	*Ka/Ks*	T(MYA)
*VrPHO1; H4*	*VrPHO1; H5*	0.16	0.75	0.21	61.48
*GmPHO1; H1*	*VrPHO1; H4*	0.13	0.65	0.21	106.61
*GmPHO1; H2*	*VrPHO1; H2*	0.1	0.28	0.36	45.65
*GmPHO1; H4*	*VrPHO1; H4*	0.13	0.65	0.19	107.23
*GmPHO1; H5*	*VrPHO1; H4*	0.06	0.37	0.16	60.68
*GmPHO1; H12*	*VrPHO1; H1*	0.04	0.22	0.17	36.22
*GmPHO1; H14*	*VrPHO1; H1*	0.03	0.2	0.14	32.92

## Data Availability

Mung bean genome-related data were obtained from the Legume Information System, and the genome ID version is vigra.VC1973A.gnm7.

## Supplementary Materials

The following supporting information can be downloaded at: https://www.mdpi.com/article/10.3390/genes17010025/s1, Table S1. Primer used for *VrPHO1* expression analysis. Table S2. Colinearity analysis of *VrPHO1*, *AtPHO1* and *GmPHO1* genes.
